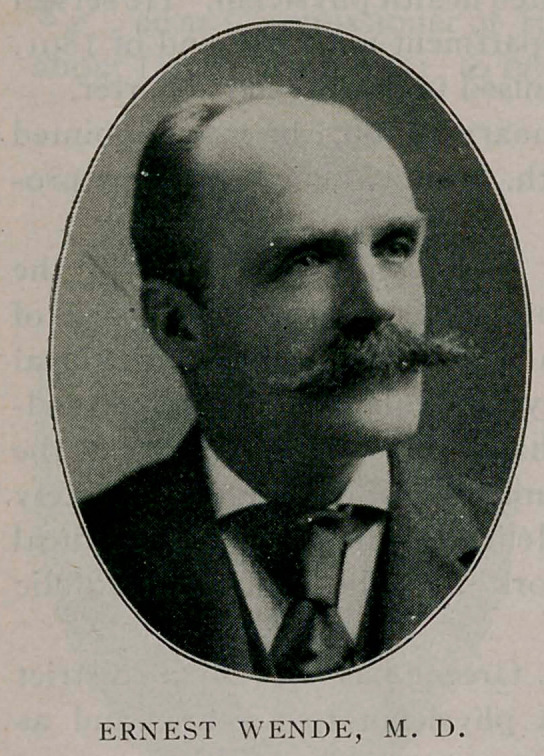# Dr. Wende’s Retirement from Office

**Published:** 1902-01

**Authors:** 


### Dr. Wende's Retirement from Office.

DR. ERNEST WENDE, who has held the office of Commissioner of Health for the City of Buffalo for ten years retired therefrom January 1, 1902. He was appointed first by Mayor Bishop, a democrat, for five years, beginning January 1, 1892, and second by Mayor Jewett, a republican, for another term of five years, beginning January 1, 1897. Mayor Knight, also a republican, declined to reappoint him for a third term, so he goes back to practise and teach the specialty of dermatology, in which he has already made for himself a conspicuous reputation.

Dr. Wende's reputation as a fearless and scientific health officer is a household word in Buffalo, and he is known abroad for the many methods he originated, and which made this city famous for its low death-rate, as well as its comparative freedom from epidemic disease. These methods are so well known that it is unnecessary to repeat them here, but they may be classed as having brought a maximum of results with a minimum of expenditure.

ERNEST WENDE, M. D.

The leading medical journals throughout the country have made extended comments upon the priniciple of keeping the health department of municipalities separated from politics, deploring the action of Buffalo's Mayor-elect in refusing to reappoint Dr. Wende, and otherwise making strenuous effort to draw the line between officials that strive to promote health and prevent disease, and other officers, and to prevent their falling a prey to the greed of the spoilsman of whatsoever party. We have not space to print even excerpts in this issue. They make interesting reading, however, and we commend them to the party managers.

Dr. Wende retires with the best wishes of his professional confrères of whatsoever party or sect, and in the consciousness of a duty faithfully performed; and these, after all, are better than further service to a thankless community, and is a reward more lasting than gold.

## Figures and Tables

**Figure f1:**